# Two NIS1-like proteins from apple canker pathogen (*Valsa mali*) play distinct roles in plant recognition and pathogen virulence

**DOI:** 10.1007/s44154-021-00031-0

**Published:** 2022-01-17

**Authors:** Jiajun Nie, Wenjing Zhou, Yonghui Lin, Zhaoyang Liu, Zhiyuan Yin, Lili Huang

**Affiliations:** 1 State Key Laboratory of Crop Stress Biology for Arid Areas, College of Plant Protection, Northwest A&F University, 3 Taicheng Road, Yangling, 712100 Shaanxi China; 2 College of Plant Protection, China Agricultural University, Beijing, 100193 China

**Keywords:** *Valsa mali*, Effector protein, Cell death, Plant immunity, Virulence

## Abstract

Conserved effectors produced by phytopathogens play critical roles in plant-microbe interactions. NIS1-like proteins represent a newly identified family of effectors distributed in multiple fungal species. However, their biological functions in a majority of pathogenic fungi remain largely elusive and require further investigation. In this study, we characterized two NIS1-like proteins VmNIS1 and VmNIS2 from *Valsa mali*, the causal agent of apple *Valsa* canker. Both of these two proteins were predicted to be secreted. Using agroinfiltration, we found that VmNIS1 induced intense cell death, whereas VmNIS2 suppressed INF1 elicitin-triggered cell death in *Nicotiana benthamiana*. Treatment of *N. benthamiana* with VmNIS1 recombinant protein produced by *Escherichia coli* activated a series of immune responses and enhanced plant disease resistance against *Phytophthora capsici*. In contrast, VmNIS2 suppressed plant immune responses and promoted *P*. *capsici* infection when transiently expressed in *N. benthamiana*. Both *VmNIS1* and *VmNIS2* were shown to be highly induced at late stage of *V. mali* infection. By individually knocking out of these two genes in *V. mali*, however, only VmNIS2 was shown to be required for pathogen virulence as well as tolerance to oxidative stress. Notably, we further showed that C-terminal extension of VmNIS1 was essential for plant recognition and VmNIS2 may escape plant detection via sequence truncation. Our data collectively indicate that VmNIS1 and VmNIS2 play distinct roles in plant recognition and pathogen virulence, which provided new insights into the function of NIS1-like proteins in plant-microbe interactions.

**Supplementary Information:**

The online version contains supplementary material available at 10.1007/s44154-021-00031-0.

## Introduction

Unlike animals, plants are sessile and lack a circulating adaptive immune system to combat pathogen attacks. Instead, they have evolved a roughly two-tiered interconnected immune signaling (Dodds and Rathjen, 2010; Jones and Dangl, 2006). As the first part of this, plants exploit cell surfaced-localized immune receptors to detect the pathogen-associated molecular patterns (PAMPs), leading to pattern-triggered immunity (PTI) (Boller and Felix, 2009). To successfully establish infection, the pathogens must block PTI at first, for which they deploy diverse effectors to manipulate host immunity (Giraldo and Valent, 2013; Toruño et al., 2016). As another part of plant immune signaling, intracellular receptors are employed for perception of so-called effectors, resulting in more potent resistance responses that provide effector-triggered immunity (ETI) (Cui et al., 2015; Lolle et al., 2020). Nevertheless, in turn, successful pathogens evolve new effectors to counteract ETI, making the plant-pathogen interactions an endless game of hide-and-seek (Deslandes and Rivas, 2012; Martel et al., 2021).

Typically, effectors are species- or lineage-specific. For example, the well characterized effector AvrPiz-t from *Magnaporthe oryzae* is specific to *Magnaporthe* spp. (Park et al., 2016); the apoplast effector Pep1 secreted by *Ustilago maydis* only distributes in *Ustilago* spp. (Hemetsberger et al., 2015); and the cytoplasmic effector Avr3a originally identified in *Phytophthora infestans* is uniquely confined to *Phytophthora* spp. (Li et al., 2019). However, there are emerging numbers of essential effectors characterized that are broadly conserved. One of the best-known examples is necrosis- and ethylene-inducing-like proteins (NLPs), which are widely distributed across bacteria, fungi, and oomycetes (Gijzen and Nürnberger, 2006; Oome and Van den Ackerveken, 2014). A few studies have shown that NLPs participate in pathogen virulence associated with phytotoxic activity, or play roles in other process such as fungal vegetable growth and conidiation (Amsellem et al., 2002; Ottmann et al., 2009; Santhanam et al., 2013). Other well-defined conserved effectors include the glycoside hydrolase 12 proteins, the fungal LysM effectors, and cerato-platanin (CP) family proteins. Among them, XEG1 from *Phytophthora sojae* (Ma et al., 2015), Ecp6 from *Cladosporium fulvum* (Bolton et al., 2008; De Jonge et al., 2010), and SsCP1 from *Sclerotinia sclerotiorum* (Yang et al., 2018) are respectively representative ones, all of which have been demonstrated to be virulence-essential.

Recently, a small secreted protein NIS1 (also referred to as CoNIS1), homologues of which are widely spread in ascomycetes and basidiomycetes fungi, has been characterized as another conserved effector (Irieda et al., 2019). Being able to trigger cell death in the model plant *N. benthamiana*, CoNIS1 and ChNIS1 were originally identified in the pathogenic fungi *Colletotrichum orbiculare* and *C. higginsianum* (Yoshino et al., 2012). Intriguingly, despite their cell death-inducing activity, CoNIS1 and ChNIS1 have also been shown to suppress INF1 elicitin-triggered cell death and immune responses induced by multiple PAMPs, thereby contributing to pathogen infection (Irieda et al., 2019). Thus far, only several NIS1-like proteins have been cloned. MoNIS1 from *M. oryzae* exhibits no cell death-inducing activity, but it inhibits plant innate immunity and is indispensable for pathogen virulence (Irieda et al., 2019; Yoshino et al., 2012). CtNIS1 from the root endophyte *C. tofieldiae* suppresses PTI responses, which may help to establish a beneficial interaction with *Arabidopsis* host (Irieda et al., 2019). FvNIS1 in *Fusarium virguliforme* was identified as a phytotoxic effector, for its capacity to cause cell death symptom in soybean host (Chang et al., 2016). Apart from these, however, little is known about this family of effectors in other pathogenic fungal species.

Apple *Valsa* canker caused by the weakly parasitic fungus *Valsa mali* has emerged globally as a devasting disease of apple, particularly, in East Asia (Togashi, 1925; Uhm and Sohn, 1995; Wang et al., 2011; Wang et al., 2020). For lacking enough knowledge on the pathogenicity determinants of *V. mali*, developing efficient controlling strategies for this disease remains a challenge. Genome analysis of *V. mali* has revealed that more than 700 effectors are produced, which may facilitate its infection on apple host (Yin et al., 2015). Consistently, several virulence-related effectors have subsequently been elucidated, including the *V. mali*-specific VmEP1 (Li et al., 2015) and VmPxE1 (Zhang et al., 2018), as well as the conserved Hce2 domain-containing proteins (Zhang et al., 2019). However, a vast majority of *V. mali* effectors remain uncharacterized.

Here, we focused on the conserved NIS1-like proteins in *V. mali*. As reported, *V. mali* possesses two homologues of NIS1 (Irieda et al., 2019). In this study, we successfully cloned these two genes, *VM1G_07228* and *VM1G_03758*, and designated them as *VmNIS1* and *VmNIS2*, respectively. We found that VmNIS1 but not VmNIS2 exhibited cell death-inducing activity, however, VmNIS2 but not VmNIS1 apparently suppressed INF1-triggered cell death in *N. benthamiana*. Further analysis revealed that VmNIS1 is an elicitor of plant immunity and is dispensable for *V. mali* virulence. Whereas, VmNIS2 acts as an effector that inhibits plant immune responses and contributes to *V. mali* full virulence. These results demonstrated that VmNIS1 and VmNIS2 from the pathogenic fungus *V. mali* play distinct roles in plant recognition and pathogen virulence. At the meanwhile, this work provided new insights into the functions of the conserved NIS1-like proteins.

## Results

### VmNIS1 and VmNIS2 are distantly related NIS1-like proteins

**Fig. 1:**
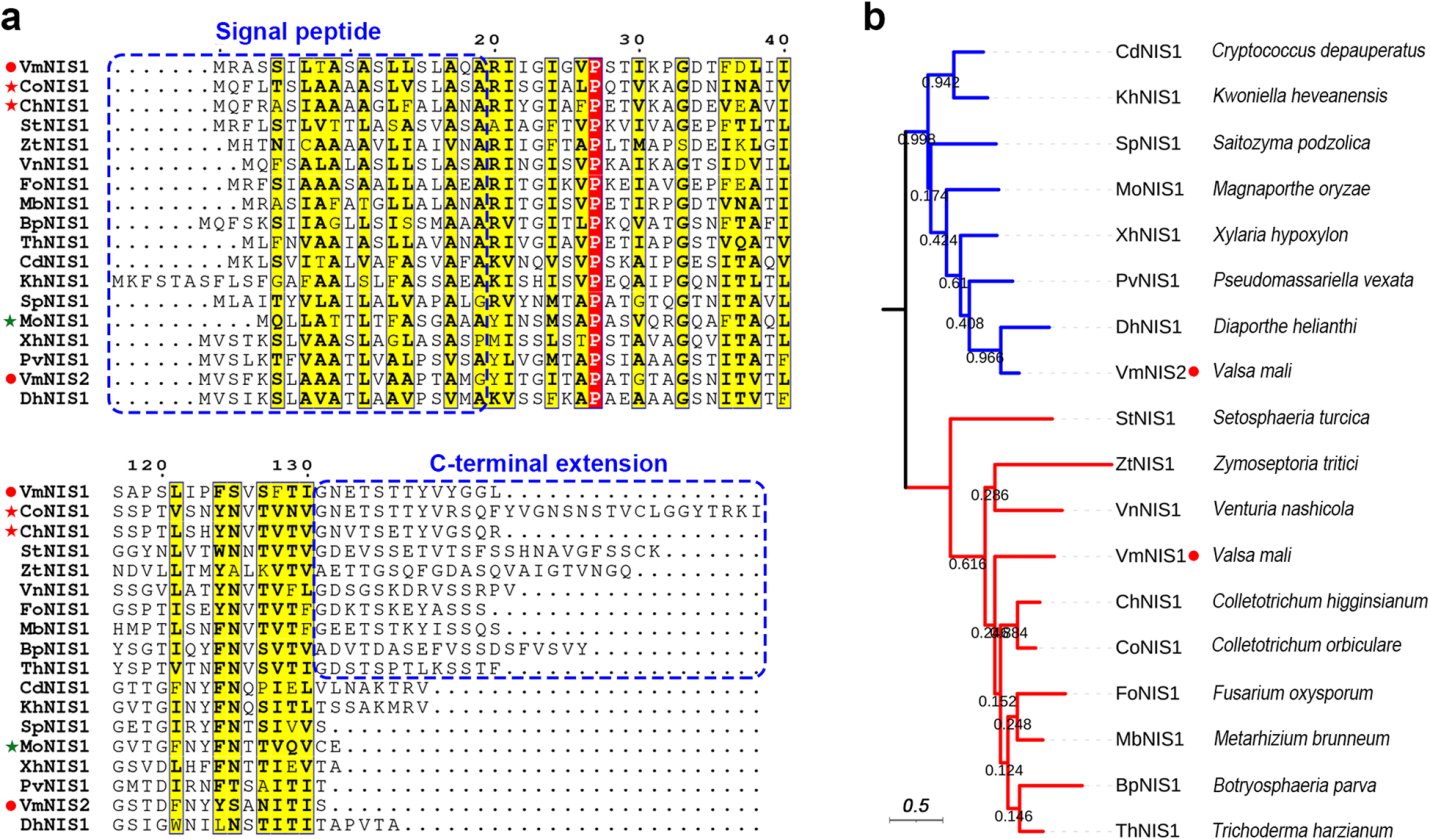
VmNIS1 and VmNIS2 are distantly related. **a** Multiple sequence alignment of VmNIS1, VmNIS2 and their homologous sequences by ClustalW. VmNIS1 and VmNIS2 from *Valsa mali*, CoNIS1 from *Colletotrichum orbiculare*, ChNIS1 from *C. higginsianum*, MoNIS1 from *Magnaporthe oryzae*, StNIS1 from *Setosphaeria turcica*, ZtNIS1 from *Zymoseptoria tritici*, VnNIS1 from *Venturia nashicola*, FoNIS1 from *Fusarium oxysporum*, MbNIS1 from *Metarhizium brunneum*, BpNIS1 from *Botryosphaeria parva*, ThNIS1 from *Trichoderma harzianum*, CdNIS1 from *Cryptococcus depauperatus*, KhNIS1 from *Kwoniella heveanensis*, SpNIS1 from *Saitozyma podzolica*, XhNIS1 from *Xylaria hypoxylon*, PvNIS1 from *Pseudomassariella vexata* and DhNIS1 from *Diaporthe helianthin* were selected for analysis. VmNIS1 and VmNIS2 are indicated by red circles. The blue dashed squares separately represent the predicted signal peptides and the extended region among selected sequences. Partial results of the alignment were shown. **b** Maximum-likelihood phylogenetic analysis of selected NIS1-like proteins showing that VmNIS1 and VmNIS2 are clustered into two distinct clades. Bootstrap support value for each branch is indicated. VmNIS1 and VmNIS2 are indicated by red circles. Red and green stars respectively indicate the proteins with and without cell death-inducing activity

To functionally characterize VmNIS1 and VmNIS2, we firstly cloned their corresponding coding genes from *V. mali* cDNA library. Sequence analysis showed that both of them carry a predicted signal peptide (SP) at N-terminus (Fig. 1a), indicating they are probably secreted. Except for this, no any known domains could be found among them. Multiple sequence alignment revealed that VmNIS1 and VmNIS2 share a moderate similarity with their homologues such as the reported CoNIS1, ChNIS1 and MoNIS1 (Fig. 1a). Interestingly, VmNIS1, CoNIS1, ChNIS1, and another 7 selected sequences including StNIS1 from *Setosphaeria turcica*, ZtNIS1 from *Zymoseptoria tritici*, VnNIS1 from *Venturia nashicola*, FoNIS1 from *Fusarium oxysporum*, MbNIS1 from *Metarhizium brunneum*, BpNIS1 from *Botryosphaeria parva* and ThNIS1 from *Trichoderma harzianum* are differentially extended at C-terminus (Fig. 1a). Consistently, phylogenetic analysis showed these sequences are exclusively clustered in the same clade (Fig. 1b). In comparison, VmNIS2, MoNIS1, and 6 other selected sequences seem to be variously truncated at C-terminus and are all clustered in a more distant clade (Fig. 1a, b). Therefore, though both being NIS1-like proteins, VmNIS1 and VmNIS2 are distantly related.

### VmNIS1 induces, while VmNIS2 suppresses cell death in *N. benthamiana*

**Fig. 2:**
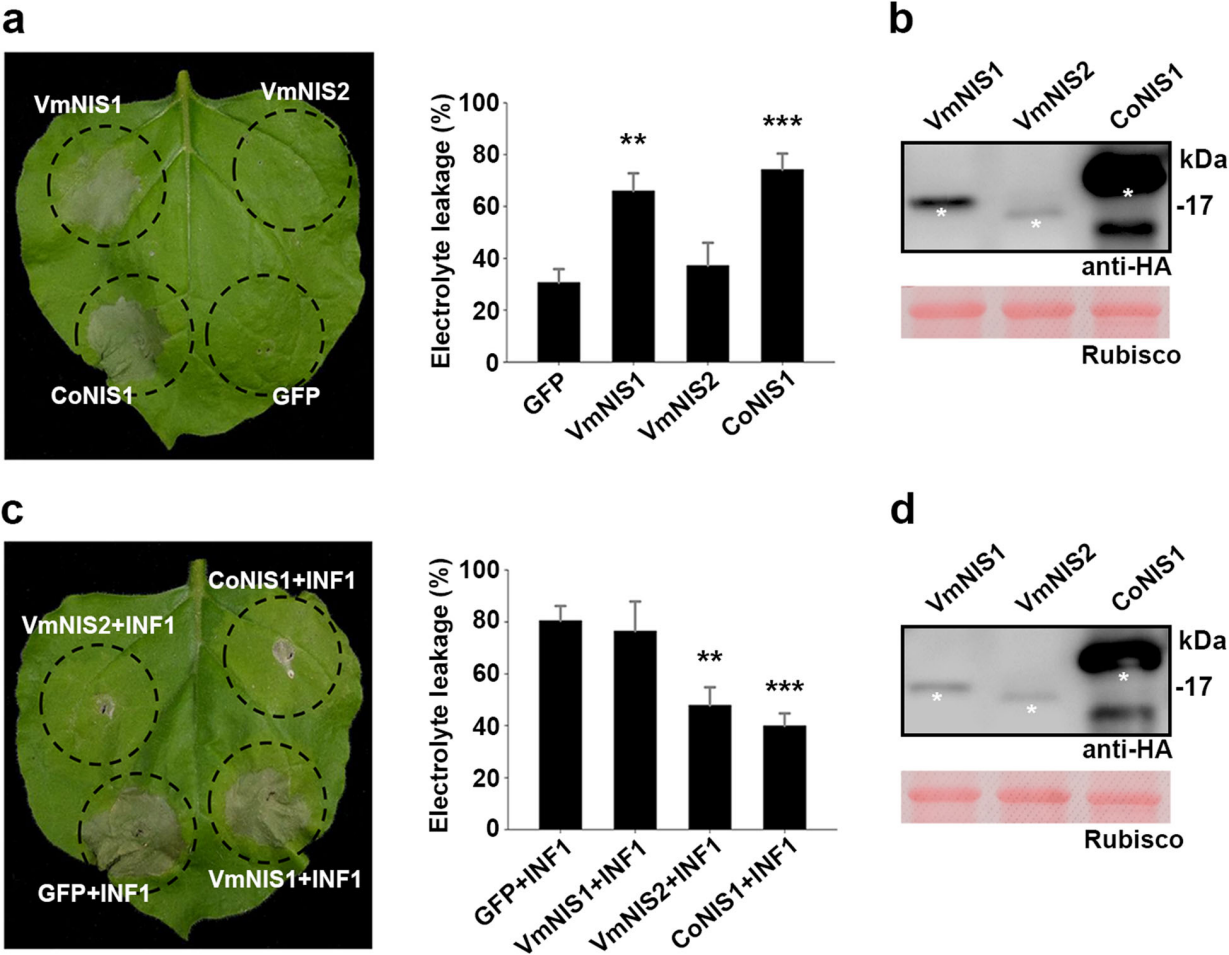
VmNIS1 induces cell death and VmNIS2 suppresses cell death in *N. benthamiana*. **a.** Representative leaves showing cell death induced by VmNIS1 in *N. benthamiana*. VmNIS1, VmNIS2, CoNIS1 and GFP were transiently expressed in *N. benthamiana* by agroinfiltration. Photographs were taken 4 days post agroinfiltration (dpa). Cell death was quantified by measuring electrolyte leakage. Values represent the means ± SD of three independent biological replicates. Differences were assessed by Student’s *t*-test. **, *P* < 0.01; ***, *P* < 0.001. **b.** Western blotting detection of VmNIS1 and VmNIS2 expressed in *N. benthamiana* leaves with anti-HA antibody. Ponceau S-stained Rubisco protein was shown as a loading control. The bans with expected size were indicated by white asterisks. **c.** Representative leaves showing VmNIS2 suppressed INF1-triggered cell death in *N. benthamiana*. VmNIS1, VmNIS2, CoNIS1 and GFP were transiently expressed in *N. benthamiana* leaves by agroinfiltration, and INF1 was agroinfiltrated in the same injection sites 24 h later. Photographs were taken 3 dpa of INF1.Cell death was quantified by measurement of electrolyte leakage. Values represent the means ± SD of three independent biological replicates. Differences were assessed by Student’s *t*-test. **, *P* < 0.01; ***, *P* < 0.001. **d.** Western blotting detection of VmNIS1 and VmNIS2 that co-expressed with INF1 in *N. benthamiana* leaves with anti-HA antibody. Ponceau S-stained Rubisco protein was shown as a loading control. The bans with expected size were indicated by white asterisks

Since NIS1-like proteins frequently trigger plant cell death, we examined whether VmNIS1 and VmNIS2 exhibited such characteristics. By transient expression in *N. benthamiana*, we found that VmNIS1, together with CoNIS1 (positive control) elicited strong cell death 5 days post agroinfiltration (dpa) (Fig. 2a, b). In contrast, VmNIS2 and GFP (a negative control) failed to cause obvious cell death, which were further confirmed by measurement of electrolyte leakage. To be noted, VmNIS1 without SP (VmNIS1^ΔSP^) showed no cell death-inducing activity in *N. benthamiana*, whereas adding the SP of pathogenesis-related protein 1(PR1) to VmNIS1^ΔSP^ ((PR1)SP-VmNIS1^ΔSP^) recovered the capacity to trigger cell death (Fig. S1a). More importantly, the full length VmNIS1 and (PR1)SP-VmNIS1^ΔSP^ but not VmNIS1^ΔSP^, can be successfully detected in the apoplastic fluid from *N. benthamiana* leaves transiently expressing these proteins (Fig. S1b), indicating that VmNIS1 is most likely targeted by SP to the apoplast space. As NIS1-like proteins are also known to suppress INF1 elicitin-triggered cell death, we next tested whether VmNIS1 and VmNIS2 worked similarly. As was shown, VmNIS2 and CoNIS1 clearly inhibited INF1-triggered cell death when expressed in *N. benthamiana*, however, VmNIS1 and the GFP control did not cause obvious alterations to this (Fig. 2c, d). Remarkably, transient expression of VmNIS2 also blocked VmNIS1-trigged cell death in *N. benthamiana* (Fig. S2). These results collectively indicate that VmNIS1 but not VmNIS2 can trigger plant cell death, whereas VmNIS2 but not VmNIS1 can suppress plant cell death.

### VmNIS1 triggers immune responses in *N. benthamiana*

**Fig. 3:**
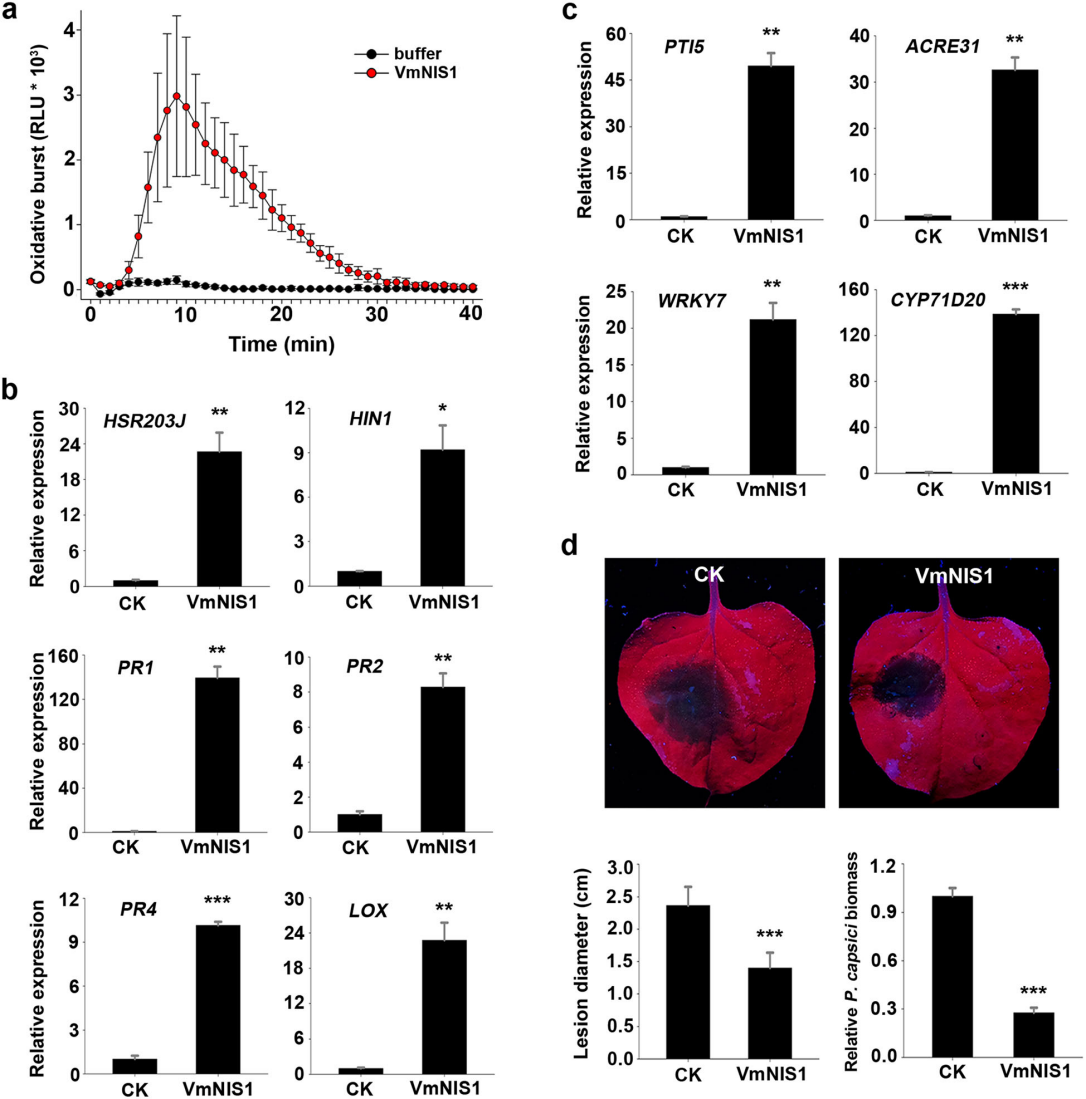
VmNIS1 triggers immune responses in *N. benthamiana*. **a.** Oxidative burst in *N. benthamiana* leaves triggered by purified VmNIS1 protein. ROS production represented by relative luminescence units (RLU) was measured through treatment with 1 μM purified VmNIS1 protein. **b.** Transcript accumulation of *HSR203J*, *HIN1*, *PR1*, *PR2*, *PR4* and *LOX* in *N. benthamiana* leaves treated with 1 μM purified VmNIS1 protein for 6 h. Transcript levels of tested genes were determined by RT-qPCR, with *NbActin* serving as the internal reference. Values represent the means ± SD of three biological replicates. Differences were assessed by Student’s *t*-test. *, *P* < 0.05; **, *P* < 0.01; ***, *P* < 0.001. **c.** Transcript accumulation of PTI marker genes *PTI5*, *ACRE31*, *WRKY7* and *CYP71D20* in *N. benthamiana* leaves treated with 1 μM purified VmNIS1 protein for 6 h. Transcript levels of these genes were determined by RT-qPCR. *NbActin* was used as the internal reference. Values represent the means ± SD of three biological replicates. Differences were assessed by Student’s *t*-test. **, *P* < 0.01; ***, *P* < 0.001. **d.** VmNIS1 protein treatment enhanced *N. benthamiana* resistance against *Phytophthora capsici*. *N. benthamiana* leaves were pretreated with 1 μM purified VmNIS1 protein for 12 h before inoculation of *P. capsici*. Representative leaves showing disease lesions were taken 36 h post inoculation under a UV light. Lesion diameters were calculated from six biological replicates. Relative biomass showing the ratios of *P. capsici* to *N. benthamiana* DNA was determined by RT-qPCR. Values represent the means ± SD. Differences were assessed by Student’s *t*-test. ***, *P* < 0.001

Pathogen-derived cell death proteins are often elicitors of plant immunity, such as XEG1 in *P. sojae* and VmE02 in *V. mali* (Ma et al., 2015; Nie et al., 2019). The observation that VmNIS1 induced cell death in *N. benthamiana* prompted us to test its ability to trigger plant immune responses. For this, VmNIS1 recombinant protein was produced by *E. coli* (Fig. S3), and immune hallmarks including ROS burst, and activation of defense-related genes were tested. Luminol-based chemiluminescence assays illustrated that, 1 μM purified VmNIS1 protein induced a high level of ROS in *N. benthamiana*, reaching a peak at 10 min upon elicitation, whereas the buffer control remained at basal levels all along (Fig. 3a). Reverse transcription-quantitative PCR (RT-qPCR) analysis showed that, a series of immune-related marker genes in *N. benthamiana* were dramatically activated after treatment with VmNIS1 protein for 6 h. Those include two hypersensitive-specific genes *HSR203J* and *HIN1* (Pontier et al., 1994; Takahashi et al., 2004), four pathogenesis-related genes *PR1*, *PR2*, *PR4* and *LOX* (Asai and Yoshioka, 2009; Dean et al., 2005; Rodriguez et al., 2014) (Fig. 3b), as well as four selected PTI marker genes *PTI5*, *Acre31*, *WRKY7* and *Cyp71D20* (Heese et al., 2007; McLellan et al., 2013) (Fig. 3c). These data suggest that VmNIS1 can be recognized and trigger potent innate immune responses in *N. benthamiana.*

To test whether VmNIS1-activated immune responses could confer plant disease resistance, we inoculated *N. benthamiana* with *Phytophthora capsici*, an oomycete pathogen that shares broad-host-ranges. As expected, we found that plant leaves pretreated with 1 μM VmNIS1 were more resistant to *P. capsici* compared with those treated with buffer control (Fig. 3d). Consistently, the average lesion diameters and relative *P. capsici* biomass on leaves treated with VmNIS1 were significantly decreased (Fig. 3d). Hence, VmNIS1 can promote plant disease resistance.

### VmNIS2 suppresses immune responses in *N. benthamiana*

**Fig. 4:**
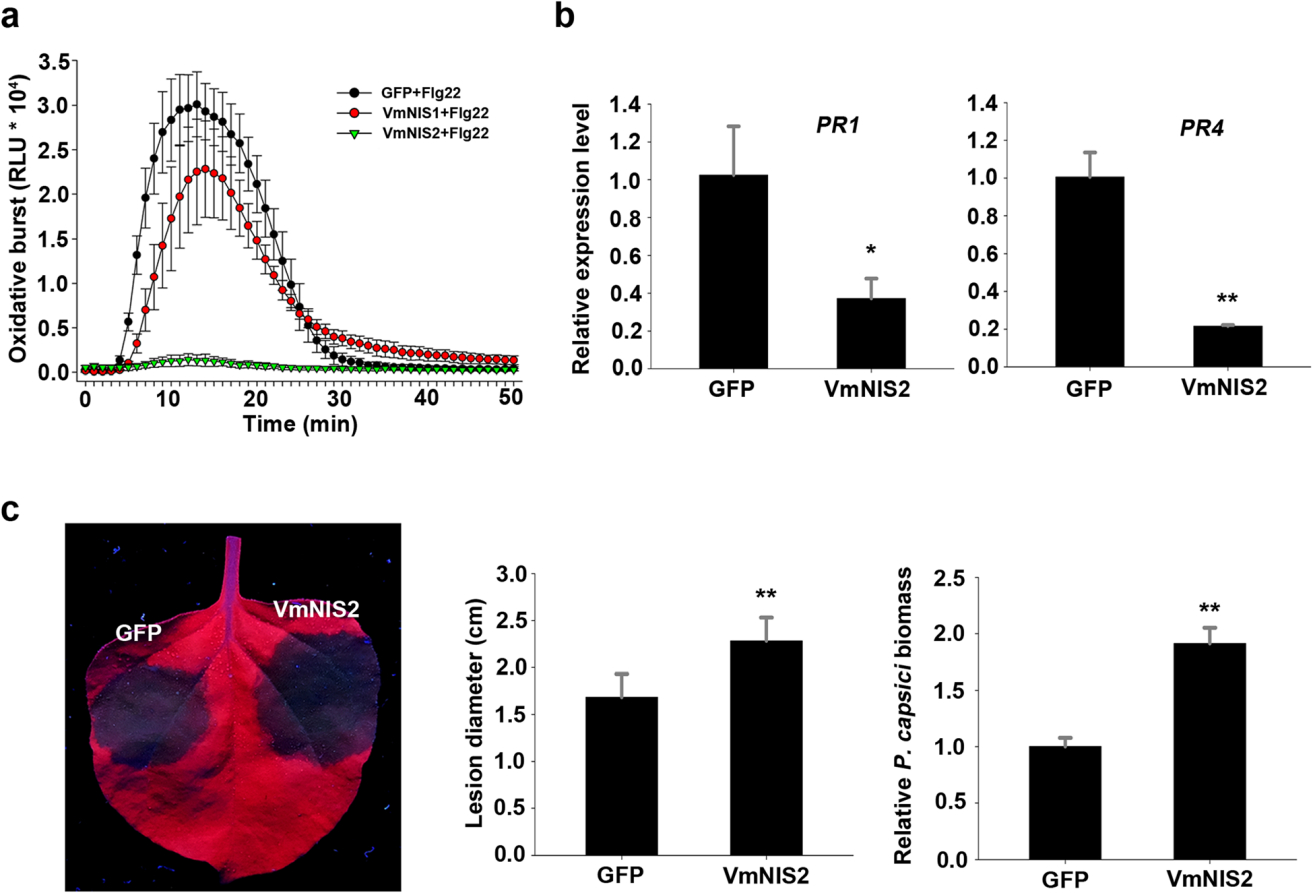
VmNIS2 suppresses immune responses in *N. benthamiana*. **a.** Influence of flg22-elicited oxidative burst by VmNIS1 and VmNIS2 in *N. benthamiana*. VmNIS1, VmNIS2 or GFP control were transiently expressed in *N. benthamiana* leaves. ROS production represented by RLU was measured by treatment with 1 μM flg22 peptide. **b.** Transcript accumulation of *PR1* and *PR4* in *N. benthamiana* transiently expressing VmNIS2 or GFP control. Transcript levels of the two genes were determined by RT-qPCR, with *NbActin* serving as the internal reference. Values represent the means ± SD of three biological replicates. Differences were assessed by Student’s *t*-test. *, *P* < 0.05; **, *P* < 0.01. **c.** Transient expression of VmNIS2 promoted *P. capsici* infection on *N. benthamiana*. The leaves were agroinfiltrated with VmNIS2 or GFP control 2 d before inoculation. Representative leaves showing *P. capsici*-caused lesions were taken 36 h post inoculation under a UV light. Lesion diameters were calculated from six biological replicates, and relative *P. capsici* biomass was determined by RT-qPCR. Values represent the means ± SD. Differences were assessed by Student’s *t*-test. **, *P* < 0.01

Unlike VmNIS1, VmNIS2 exhibited no cell death-inducing activity, but rather inhibited cell death triggered by INF1 elicitin when transiently expressed in *N. benthamiana* (Fig. 2a, c). To verify whether VmNIS2 can suppress plant immune responses, we measured ROS burst induced by flg22, a typical PAMP in bacteria (Chinchilla et al., 2007), and determined the transcript accumulation of immune marker genes by transient expression in *N. benthamiana*. It was shown that, compared with GFP control, VmNIS2 markedly blocked flg22-triggered ROS generation, whereas VmNIS1 only caused a slight decrease of this (Fig. 4a). In addition, as revealed by RT-qPCR analysis, the transcript levels of *PR1* and *PR4* were greatly attenuated in *N. benthamiana* leaves expressing VmNIS2 (Fig. 4b). We further examined whether VmNIS2 can impair plant disease resistance. As shown in Fig. 4c, *N. benthamiana* leaves transiently expressing VmNIS2 was more susceptible to *P. capsici*, compared with that expressing GFP. This result was further confirmed by quantification of lesion diameters and relative *P. capsici* biomass. Based on these above, we concluded that VmNIS2 acts as a suppressor of plant immunity.

### VmNIS2 but not VmNIS1 contributes to *V. mali* full virulence and oxidative stress tolerance

**Fig. 5:**
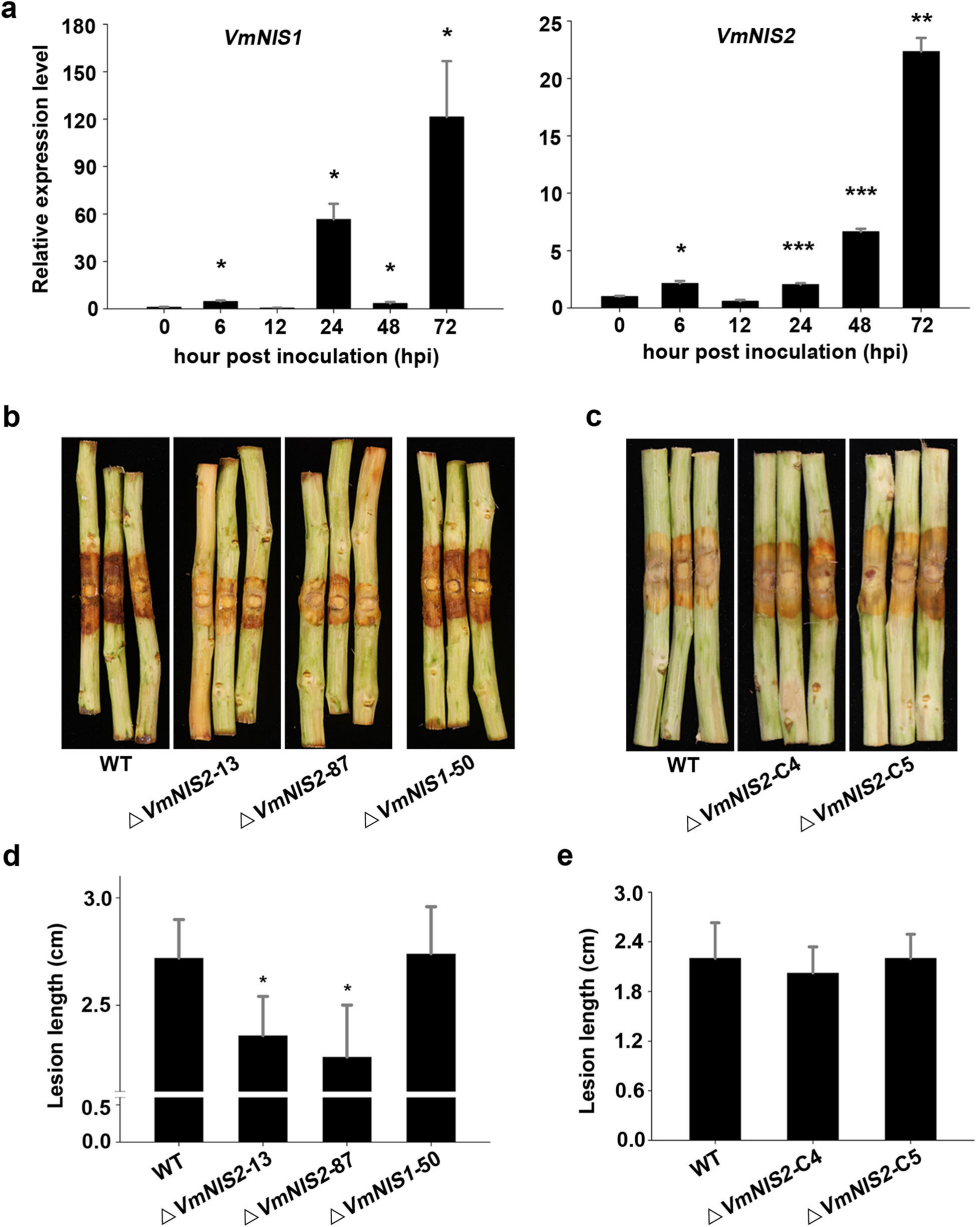
VmNIS2 contributes to *Vala mali* full virulence. **a.** Expression profiles of *VmNIS1* and *VmNIS2* during *V. mali* infection of apple host. Transcript levels of *VmNIS1* and *VmNIS2* at 0, 12, 24, 36, 48, and 72 h post inoculation (hpi) of *V. mali* wild type strain 03–8 on apple twigs were accessed by RT-qPCR, with *G6PDH* used as the internal reference. Values represent the means ± SD of three biological replicates. Differences were assessed by Student’s *t*-test. *, *P* < 0.05; **, *P* < 0.01; ***, *P* < 0.001. **b., d.** Disease lesions caused by *V. mali* wild type, *VmNIS1* deletion mutant (Δ*VmNIS1*–50), and *VmNIS2* deletion mutants (Δ*VmNIS2*–13 and Δ*VmNIS2*–87) on apple twigs. Representative photographs for infected apple twigs (**b**) were taken 3 d post inoculation (dpi). Lesion lengths (**d**) were calculated from five biological replicates. Values represent the means ± SD. Differences were assessed by Student’s *t*-test. *, *P* < 0.05. **c., e.** Disease lesions caused by *V. mali* wild type and *VmNIS2* complementation transformants (Δ*VmNIS2*-C4 and Δ*VmNIS2*-C5) on apple twigs. Representative photographs (**c**) were taken and lesion lengths (**e**) were calculated 3 dpi. Values represent the means ± SD of five biological replicates

To investigate the biological roles of VmNIS1 and VmNIS2 in *V. mali*, their expression profiles were first analyzed by RT-qPCR. Both *VmNIS1* and *VmNIS2* were activated during pathogen infection (Fig. 5a). Remarkably, their transcript levels were dramatically elevated at late stage of infection, reaching a maximum at 72 h post inoculation (hpi) (Fig. 5a), indicating they both may participate in *V. mali* infection.

To test their potential virulence roles, *VmNIS1* and *VmNIS2* were accordingly knocked out in the wild type strain 03–8 using PEG-mediated protoplast transformation. Two independent deletion mutants for *VmNIS2* (Δ*VmNIS2*–13 and Δ*VmNIS2*–87) were successfully obtained (Fig. S4). However, only one deletion mutant for *VmNIS1* (Δ*VmNIS1*–50) was generated (Fig. S4) through enormous attempts. Filamentous growth of all these mutants displayed no apparent difference with that of the wild type when cultured on PDA plates (Fig. S5). Virulence tests showed that Δ*VmNIS1*–50 infection on apple twigs was hardly affected, however, the lesion length caused by either Δ*VmNIS2*–13 or Δ*VmNIS2*–87 was significantly reduced (Fig. 5b, d). After introducing the complementing plasmids into *VmNIS2* deletion mutants (Fig. S4), comparable infection lesions with the wild type were observed (Fig. 5c, e). Altogether, these results demonstrate that VmNIS2 but not VmNIS1 contributes to *V. mali* full virulence.

**Fig. 6:**
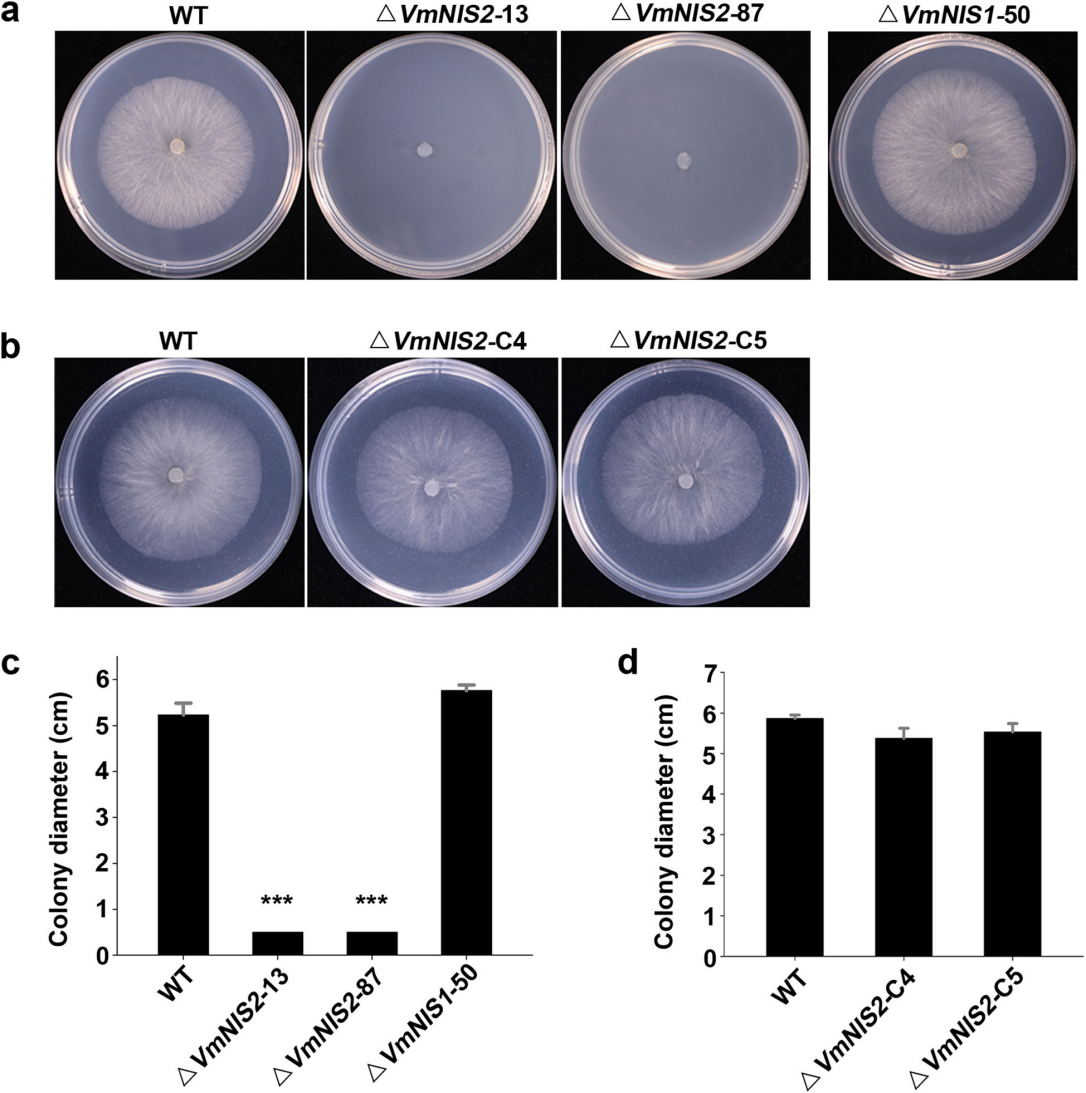
VmNIS2 is required for *V. mali* tolerance to oxidative stress. **a., c.** Filamentous growth of *V. mali* wild type, *VmNIS1* deletion mutant (Δ*VmNIS1*–50), and *VmNIS2* deletion mutants (Δ*VmNIS2*–13 and Δ*VmNIS2*–87) on PDA medium supplemented with 10 mM H_2_O_2_. Representative photographs (**a**) were taken after culturing for 48 h. Colony diameters (**c**) were calculated from nine biological replicates. Values represent the means ± SD. Differences were assessed by Student’s *t*-test. ***, *P* < 0.001. **b., d.** Filamentous growth of *V. mali* wild type and *VmNIS2* complementation transformants (Δ*VmNIS2*-C4 and Δ*VmNIS2*-C5) on PDA medium supplemented with 10 mM H_2_O_2_. Representative photographs (**b**) were taken after culturing for 48 h and colony diameters (**d**) were calculated. Values represent the means ± SD from nine biological replicates

We then tested whether the deletion mutants were sensitive to abiotic stresses like salt and oxidate. For this, we challenged them with KCl and H_2_O_2_, respectively. Both *VmNIS1* and *VmNIS2* deletion mutants grew as well as the wild type on PDA plates with 500 mM KCl (Fig. S6). Intriguingly, when treated with 10 mM H_2_O_2_, *VmNIS2* deletion mutants almost failed to colonize on the plates, whereas Δ*VmNIS1*–50 exhibited similar colony morphology as the wild type (Fig. 6a, c). On the contrary, complementation of *VmNIS2* in its deletion mutants restored the ability to grow under the same condition (Fig. 6b, d). Therefore, VmNIS2 but not VmNIS1 is required for *V. mali* tolerance to oxidative stress.

### VmNIS2 may escape plant detection via C-terminal truncation

BAK1, a common coreceptor for many cell surface-localized receptors (Yasuda et al., 2017), has been reported to be targeted by CoNIS1 and MoNIS1 for immunity manipulation (Irieda et al., 2019). Considering the distinct roles of VmNIS1 and VmNIS2 in plant recognition and *V. mali* virulence as illustrated above, we speculated that they might exhibit discrepancy in BAK1-interactions. To test this assumption, we generated mCherry-tagged VmNIS1 and VmNIS2, as well as GFP-tagged *N. benthamiana* BAK1 (NbBAK1) to perform a co-immunoprecipitation (Co-IP) assay. The VmE02 elicitor in *V. mali* (Nie et al., 2019) was used a negative control. Unexpectedly, VmNIS1 and VmNIS2 both successfully immunoprecipitated NbBAK1, and in contrast, no obvious interaction could be detected between BAK1 and VmE02 (Fig. S7). This result indicates that VmNIS1 and VmNIS2 also interact with the BAK1 coreceptor *in planta*.

**Fig. 7:**
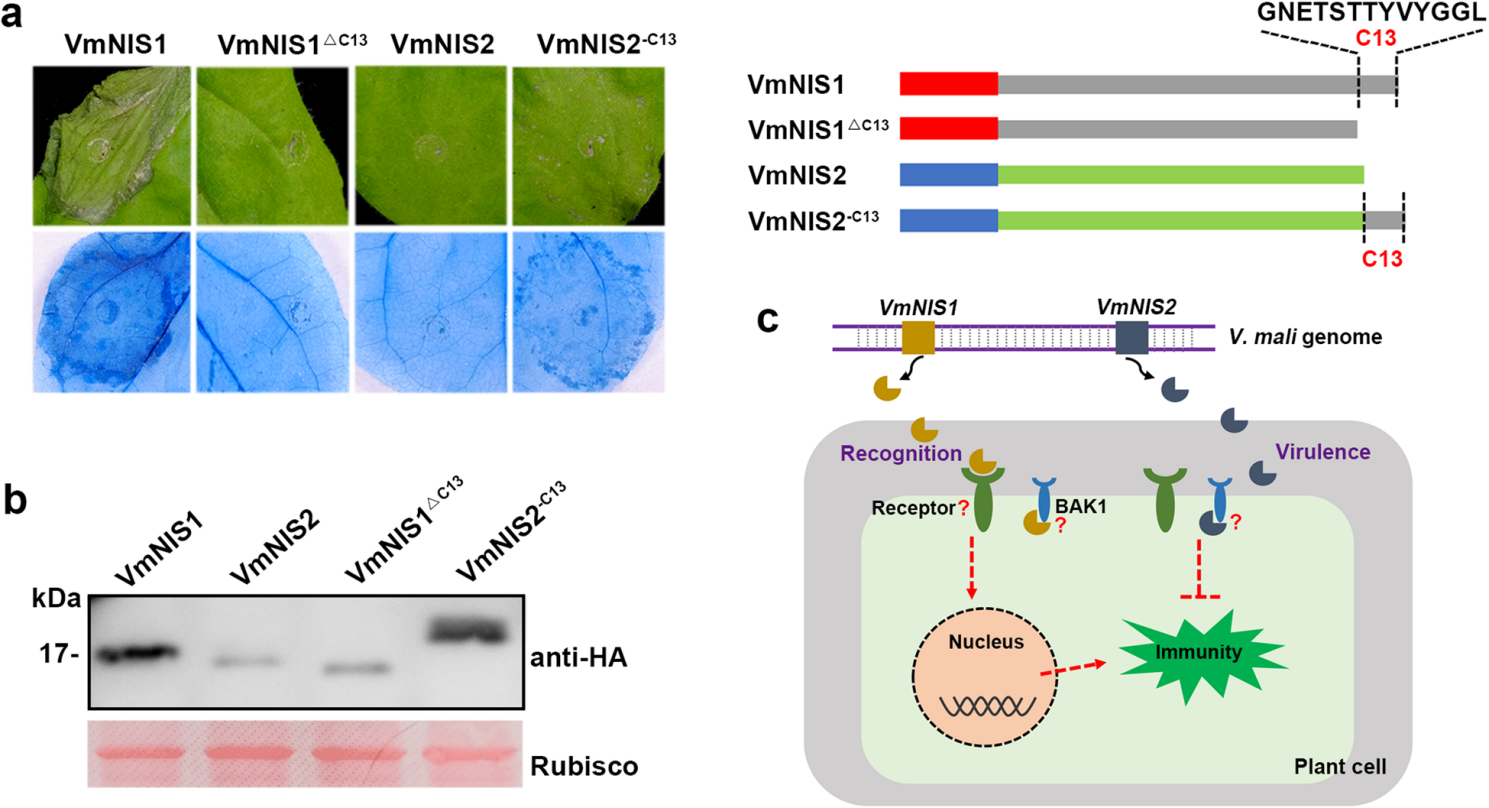
VmNIS2 may escape plant detection by C-terminal truncation. **a.** Representative leaves showing cell death induced by VmNIS1 and VmNIS2^-C13^ in *N. benthamiana*. VmNIS1, VmNIS1^ΔC13^, VmNIS2 and VmNIS2^-C13^ were transiently expressed in *N. benthamiana* by agroinfiltration. Photographs were taken 5 dpa. Cell death symptom was further visualized by trypan blue staining. A diagram showing the protein constructs is depicted. **b.** Western blotting detection of the expressed proteins with anti-HA antibody. Ponceau S-stained Rubisco protein was shown as a loading control. **c.** A proposed model for VmNIS1 and VmNIS2 function in plant-pathogen interactions. VmNIS1 is recognized by the plant via a yet-unknown receptor and activates plant immunity. VmNIS2 serves as a virulence factor that escapes plant recognition and suppresses plant immunity

Since VmNIS1 sequence is extended at C-terminus when compared with that of VmNIS2 (Fig. 1), we subsequently tested whether this variation results in their executively biological roles. To this end, we created two variants VmNIS1^ΔC13^ and VmNIS2^-C13^, with C-terminal 13 amino acid residues of VmNIS1 (C13) deleted and C13 fused to VmNIS2 at terminus, respectively. Transient expression analysis showed that VmNIS1^ΔC13^ failed to triggered apparent cell death in *N. benthamiana* as the full length VmNIS1 did (Fig. 7a, b), indicating C13 is essential for plant detection of VmNIS1. Intriguingly, in contrast to VmNIS2, VmNIS2^-C13^ induced a weak but visible cell death symptom (Fig. 7a, b), suggesting C13 may enable VmNIS2 to be recognized by *N. benthamiana*. To tested whether C13 can be directly perceived by *N. benthamiana*, we obtained the Pep13 peptide corresponding to C13 sequence and tested its immunogenic activity on *N. benthamiana*. Unfortunately, Pep13 was not capable of eliciting apparent ROS burst as the flg22 PAMP did, even when its reaction concentration increased to 10 μM (Fig. S8). These data collectively suggest that C13 of VmNIS1 is essential but not sufficient to be detected by plant, and VmNIS2 may avoid plant recognition through C-terminal truncation during evolution thereby serving as a virulence factor (Fig. 7c).

## Discussion

Conserved effectors produced by pathogens such as necrosis- and ethylene-inducing-like proteins (NLPs) and glycoside hydrolase 12 proteins play important roles in plant-microbe interactions (Ma et al., 2015; Oome and Van den Ackerveken, 2014). In this study, with the aim to explore essential virulence effectors in apple *Valsa* canker pathogen (*V. mali*), we focused on the NIS1-like proteins, a family of ‘core’ effectors that are widely spread in various fungal species (Irieda et al., 2019; Yoshino et al., 2012). Correspondingly, two NIS1-like proteins, VmNIS1 and VmNIS2 were cloned and characterized.

VmNIS1 was shown to be evolutionarily related to CoNIS1 from *Colletotrichum orbiculare* (Fig. 1b), which is identical to the results described by Irieda and colleagues (Irieda et al., 2019). Consistently, the full-length VmNIS1 as well as CoNIS1 triggered intense cell death when transiently expressed in *N. benthamiana* (Fig. 2a). Moreover, it was reported that deletion of *CoNIS1* posed no obvious effect on *C. orbiculare* virulence (Yoshino et al., 2012). Here, though *VmNIS1* was highly induced at late stage *V. mali* infection (Fig. 5a), the deletion mutant of *VmNIS1* showed nearly the same pathogenicity as the wild type (Fig. 5b, d). Therefore, VmNIS1 and CoNIS1 are to a great extent functionally conserved. A major difference between VmNIS1 and CoNIS1 locates in their discrepant capacity to suppress plant cell death and ROS burst. CoNIS1 has been demonstrated to strongly block INF1-triggered cell death and flg22-induced ROS generation (Irieda et al., 2019), however, VmNIS1 neither inhibited INF1-triggered cell death nor drastically attenuated flg22-induced ROS burst (Fig. 2c and Fig. 4a). Such a difference indicates the potential function diversity among this family of proteins.

PAMPs are considered as evolutionarily conserved molecules recognized by plant in the apoplast (Boutrot and Zipfel, 2017; Nürnberger and Brunner, 2002). Importantly, several lines of evidence suggest that VmNIS1 exhibits typical characteristics of a PAMP. First of all, VmNIS1 belongs to NIS1-like proteins that are well conserved in multiple fungi. Next, VmNIS1 recombinant protein activated a series of immune responses in *N. benthamiana*, including induction of ROS burst, elevated expression of defense-related genes, and enhanced resistance to plant disease (Fig. 3), which strongly implies that VmNIS1 is recognized by *N. benthamiana*. In particular, four selected PTI marker genes including *PTI5*, *Acre31*, *WRKY7* and *Cyp71D20* were dramatically induced by VmNIS1 treatment (Fig. 4c). For another, VmNIS1 without SP (signal peptide) was unable to elicit cell death in *N. benthamiana*, and the SP of VmNIS1 can be replaced by that of PR1 (Fig. S1). This indicates that VmNIS1 should be targeted to the apoplastic space for plant recognition, possibly by certain RLK-type receptors (Chang et al., 2016). Altogether, VmNIS1 is most likely to be recognized by *N. benthamiana* as a new PAMP among fungal species.

In contrast to VmNIS1, VmNIS2 was shown to be phylogenetically close to *M. oryzae* MoNIS1 (Fig. 1a), a NIS1-like protein that exhibits no cell death-inducing activity (Yoshino et al., 2012). Likewise, transient expression of VmNIS2 cannot induce cell death (Fig. 1b), and VmNIS2 clearly suppressed INF1-triggered cell death and flg22-induced ROS burst when expressed in *N. benthamiana* (Fig. 2c and Fig. 4a), similar to that observed for MoNIS1 (Irieda et al., 2019). Furthermore, expression of VmNIS2 in *N. benthamiana* attenuated the transcripts of immune-related marker genes and plant resistance to *P. capsici* (Fig. 4b, c). More importantly, the transcript accumulation of *VmNIS2* was considerably elevated during *V. mali* infection, and the infection of *VmNIS2* deletion mutants on apple host were significantly compromised (Fig. 5), demonstrating that VmNIS2 serves as an essential virulence factor like MoNIS1. Though *VmNIS1* transcripts were even more strongly induced during *V. mali* infection than *VmNIS1*, it displayed no obvious contribution to pathogen virulence (Fig. 5a, b). It is possible that VmNIS2 suppresses VmNIS1 activity (Fig. S2) at translational or post-translational level. Our co-immunoprecipitation assays revealed VmNIS2 interacted with the *N. benthamiana* receptor-like kinase BAK1(NbBAK1) *in planta* (Fig. S7), suggesting that it probably manipulates plant immunity in the same way as MoNIS1 (Irieda et al., 2019). Therefore, VmNIS2 is functionally close to MoNIS1. To be noted, VmNIS2 also plays a role in *V. mali* tolerance to oxidative stress apart from virulence, (Fig. 6), further supporting the functional diversity of the NIS1-like proteins.

If VmNIS1 is a PAMP perceived by plants, then VmNIS2 probably has escaped plant detection via C-terminal truncation. As displayed in Fig. 1a, VmNIS1 and CoNIS1 are differentially extended at C-terminus, compared with VmNIS2 and MoNIS1. It is possible that the C-terminal extended region is required for their recognition by certain host plants. In consistent with this hypothesis, 30 C-terminal amino acid residues of CoNIS1, which are among its extended region, have been demonstrated to be indispensable for cell death activation (Yoshino et al., 2012). Likewise, a VmNIS1 truncated variant with 13 amino acid residues deleted at C-terminus (VmNIS1^ΔC13^) also failed to trigger cell death in *N. benthamiana* (Fig. 7a). It is worth mentioning that, by adding the 13 C-terminal amino acid residues to the end of VmNIS2, the fusion protein (VmNIS2-C13) showed a weak cell death-inducing activity in *N. benthamiana* (Fig. 7a). Therefore, it is reasonable that VmNIS2 discarded the extension region during long-term evolution, thereby avoiding plant recognition and acting as an effector to promote pathogen virulence (Fig. 7c). At the meanwhile, however, we cannot exclude a possibility that VmNIS2 serves as a ‘decoy’ effector, which is reminiscent of PsXLP1 from *P. sojae* (Ma et al., 2017) and the iTALE effectors from *Xanthomonas oryzae* (Ji et al., 2016; Read et al., 2016). In addition to being perceived as a PAMP, XEG1 functions as a virulence effector in *P. sojae* and is targeted by the defense protein GmGIP1 in soybean (Ma et al., 2017). However, *P. sojae* has evolved an XEG1-like protein PsXLP1 that is truncated at C-terminus, and it tightly binds GmGIP1 to protect XEG1 from host inhibition (Ma et al., 2017). Typical TALE effectors are major virulence determinants of *Xanthomonas* but can be recognized by certain host plants that carry *R* genes encoding NLR (Nod-like receptor) proteins (Paulus et al.,^ 2017^). For counteraction, iTALE (or truncated TALE) effectors that can either avoid or suppress perception by NLRs have been evolved to promote disease (Ji et al., 2016; Read et al., 2016). In this study, both VmNIS1 and VmNIS2 can interact with the receptor-like kinase BAK1 *in planta* (Fig. S7). Nonetheless, whether they function similarly in a ‘decoy’ way needs to be further investigated. It is noteworthy that, their same interactions with BAK1 are inconsistent with their distinct biological functions observed in this study. Since VmNIS1 failed to dramatically suppress flg22-triggered ROS burst in *N. benthamiana* as VmNIS2 did (Fig. 4a), it possibly cannot inhibit BAK1 kinase activity potently, and thus its interaction with BAK1 may be inactive.

In summary, we revealed that VmNIS1 and VmNIS2 in *V. mali* play distinct roles in plant recognition and pathogen virulence. This work essentially advanced our knowledge on *V. mali*-apple interaction and provided novel insights into the function of fungal NIS1-like proteins. To better understand how these two proteins collectively work during *V. mali* infection, novel host components such as the potential cell surface immune receptor(s) targeted by them should be explored in future.

## Materials and methods

### Strains and plant materials

The *Valsa mali* strain 03–8 was cultured on potato dextrose agar (PDA) medium at 25 °C. *E. coli* strain Top10 and *Agrobacterium tumefaciens* strain GV3101were grown on lysogeny broth (LB) medium, at 37 °C and 28 °C, respectively. *Phytophthora capsici* strain LT263 was maintained on 20% (v/v) V8 juice agar at 25 °C. *N. benthamiana* seedlings and *Malus domestica* borkh. cv. ‘Fuji’ were grown in a climate chamber with 16 h photoperiod at 22 °C.

### Plasmid construction

VmNIS1 and VmNIS2 coding sequences were amplified from *V. mali* cDNA library and CoNIS1 was cloned from synthetic double-strand DNAs produced by Sangon, Inc. (Shanghai, China). INF1 and *N. benthamiana* BAK1 were cloned from previous binary vectors (Nie et al., 2021). For transient expression, purified fragments were cloned into pGR106, pCAMBIA1302, pCAMBIA1300 or pICH86988 vectors prior to mobilization into *A. tumefaciens* GV3101. For protein expression in *E. coli*, the fragments were cloned into pET28a vector. To generate gene complementation constructs, the fragments were cloned into pDL2 vector (Zhou et al., 2011). All sequences were amplified by PCR using Phusion High-Fidelity DNA Polymerase (New England Biolabs, Ipswich, MA, USA), and were subsequently ligated to vectors digested with specific restriction enzymes using ClonExpress II One-Step Cloning Kit or ClonExpress MultiS One-Step Cloning Kit (Vazyme, Nanjing, China). Primers used in this study were listed in Table S1.

### Agroinfiltration in *N. benthamiana*

*A. tumefaciens* GV3101 carrying plasmids were cultured on LB medium with appropriate antibiotics at 28 °C to an OD_600_ of ~ 1.5. The bacterial cells were then collected via centrifugation and were suspended in MES buffer (10 mM MgCl_2_, 10 mM 2-(N-morpholino) ethanesulfonic acid (MES), 200 μM acetosyringone, pH 5.7) in the dark for at least 2 h. The bacteria suspension was adjusted to a final OD_600_ of 0.6 ~ 0.8 prior to infiltration into *N. benthamiana* leaves using a needleless syringe. To examine cell death suppression, *A. tumefaciens* cells carrying full-length VmNIS2 (with signal peptide), full-length CoNIS1 or GFP were infiltrated 24 h after injection of those carrying INF1 or full-length VmNIS1 as reported (Irieda et al., 2019). Symptoms were monitored 3–5 dpa. All assays were performed on no less than six leaves from individual plant seedlings, and the experiments were repeated at least three times.

### Electrolyte leakage measurement and trypan blue staining

Cell death symptoms were quantified by either ion leakage or visualized via trypan blue staining. To measure electrolyte leakage, six disks (diameter 1 cm) were isolated from agroinfiltrated *N. benthamiana* leaves and gently placed in a tube containing 5 mL distilled water. After incubation at room temperature (RT) for 5 h, the conductivity of the bathing solution was measured using a conductivity meter (FE32 FiveEasy; Mettler-Toledo, Shanghai, China) to generate an ‘A value’. Next, the leaf disks were boiled in the bathing solution for 20 min. When it cooled to RT, the conductivity was measured again to yield a ‘B value’. Ion leakage was represented as the percentage of total released ions, i.e. (A value / B value) × 100. Assays were repeated three times.

For trypan blue staining, agroinfiltrated *N. benthamiana* leaves were detached and floated in the trypan blue staining solution (0.1% trypan blue, a 1:1:1:1:8 mixture of phenol, glycerol, lactic acid, water and ethanol). The leaves were boiled in the staining solution for 3 min and were incubated at RT for at least 12 h, followed by destaining with saturated solution of chloral hydrate. Pictures were taken using a digital camera.

### Recombinant protein expression and purification

VmNIS1 without signal peptide was cloned into pET28a vector, and the plasmid was mobilized into *E. coli* strain BL21(DE3) for expression of N-terminal His-tagged VmNIS1. Expression was induced in LB medium containing 0.2 mM isopropyl-β-D-thiogalactopyrandoside (IPTG). After incubation for 24 h at 16 °C, the bacterial cells were collected by centrifugation and washed twice with PBS buffer (20 mM Na_2_HPO_4_, 300 mM NaCl, pH 7.4). The samples were then transferred into lysing buffer (20 mM Na_2_HPO_4_, 300 mM NaCl, pH 7.4, 6 M guanidine hydrochloride, 1 mM PMSF) and incubated at RT for 1 h. The supernatant containing denatured VmNIS1 protein was obtained via sonication and centrifugation. VmNIS1 recombinant protein was purified using Ni-NTA resin (Thermo Scientific, Waltham, MA, USA) according to the manufacturer’s instructions, and was further refolded by stepwise dialyzing.

### RNA extraction and RT-qPCR analysis

Total RNA extraction and cDNA synthesis were performed using Quick RNA isolation Kit (Huayueyang, Beijing, China) and RevertAid First Strand cDNA Synthesis Kit (Thermo Scientific, Waltham, MA, USA) following the manufacturer’s instructions, respectively. Reverse transcription-quantitative polymerase chain reaction (RT-qPCR) was performed using ChamQ Universal SYBR qPCR Master Mix (Vazyme, Nanjing, China) in a LightCycler 96 System (Roche, Germany). *G6PDH* (Yin et al., 2013) was used as an internal reference to normalize gene expressions in *V. mali*, and *NbActin* (Sainsbury and Lomonossoff, 2008) was used as an internal reference to normalize gene expressions in *N. benthamiana*. Relative expression levels were analyzed through the 2^-ΔΔCT^ method (Livak and Schmittgen, 2001).

### ROS burst measurement

To examine ROS burst elicited by VmNIS1 recombinant protein, Pep13 (peptide derived from VmNIS1) or flg22 PAMP, leaf disks (diameter 0.5 cm) were collected from healthy *N. benthamiana* leaves using a cork-borer set. To examine suppression of flg22 PAMP-induced ROS burst, leaf disks were collected from *N. benthamiana* leaves transiently expressing VmNIS1, VmNIS2 or GFP. The disks were put in a 96-well plate and floated in ultra-pure distilled water overnight. Prior to luminescence measurement with a Varioskan LUX multimode microplate reader (Thermo Scientific), water in each well of the plate was replaced with 100 μL reaction solution containing 100 μM luminol (Solarbio, Beijing, China), 20 μg mL^−1^peroxidase (Solarbio, Beijing, China), and 1 μM VmNIS1 purified protein or flg22 peptide. Eight biological replicates were used each time. The experiments were repeated three times with similar results. Pep13 peptide was synthesized by Sangon Biotech (Shanghai, China), and flg22 peptide was purchased from Genscript Biotech (Nanjing, China).

### Transformants generation and pathogen inoculation assays

Both gene knocking out and complementation transformants were generated by PEG-mediated protoplast transformation based on a previously described method (Li et al., 2015). A schematic representation showing the strategy for gene deletion and transformants verification was depicted (Fig. S4a). For virulence tests, apple twigs were detached and inoculated with the transformants or *V. mali* wild type as described (Wei et al., 2010). For *P. capsici* infection, *N. benthamiana* leaves were infiltrated with 1 μM VmNIS1 purified protein or *A. tumefaciens* cells carrying VmNIS2 prior to inoculation. 12 h post purified protein treatment or 24 h post agroinfiltration, fresh *P. capsici* mycelial plugs (diameter 0.5 cm) collected from V8 juice agar plates were inoculated on the back of these leaves. The leaves were then put in a transparent box to keep high humidity and placed in a growth chamber at 25 °C under dark conditions. Average lesion diameters were measured 36 hpi, and relative *P. capsici* biomass was quantified using RT-qPCR as described (Yu et al., 2012).

### Co-immunoprecipitation and western blotting

*N. benthamiana* leaves transiently expressing indicated proteins were harvested 36–48 h post agroinfiltration, and lysing buffer (50 mM Tris, 150 mM NaCl, 1 mM ethylenediaminetetraacetic acid (EDTA), 5% glycerol, 0.5% TritonX-100, 5 mM dithiothreitol (DTT), 1 mM phenylmethanesulfonyl fluoride (PMSF), and 1% proteinase inhibitor cocktail, pH 7.5) was used to extract total proteins. The apoplastic fluid was extracted by infiltration-centrifugation method as previously described (Nie et al., 2019). Immunoprecipitations were performed using GFP-Trap A beads (Chromotek, Planegg-Martinsried, Germany) following the manufacturer’s instructions. Protein samples were subjected to western blotting analysis with anti-GFP (Abways, Shanghai, China), anti-HA (Abcam, Cambridge, UK, ab18181), anti-mCherry (Sungenebiotech, Tianjin, China) or anti-His (Abways, Shanghai, China) monoclonal antibodies, as well as goat-anti mouse IgG secondary antibody (Abways, Shanghai, China). The blots were detected using ECL substrate kit (GE Healthcare, RPN2235).

### Bioinformatics analysis

Signal peptide prediction was performed through the online SignalP 5.0 server (http://www.cbs.dtu.dk/services/SignalP/). Multiple sequence alignment was carried out by the alignment tool ClustalW (https://www.genome.jp/tools-bin/clustalw), and ESPript (http://espript.ibcp.fr/ESPript/ESPript/) was used for alignment visualization. A maximum-likelihood tree was constructed using MEGA X (Kumar et al., 2018) and optimized by the website tool iTOL (https://itol.embl.de/).

### Accession numbers

Sequences in this study can be found in the GenBank data library under the following accession nos. VmNIS1 (KUI71026.1), VmNIS2 (KUI68467.1), CoNIS1 (N4VG36.1), ChNIS1 (XP_018157746.1), MoNIS1 (XP_003709116.1), StNIS1 (XP_008025198.1), ZtNIS1 (SMQ54144.1), VnNIS1 (TID19999.1), FoNIS1 (XP_018248187.1), MbNIS1 (XP_014544275.1), BpNIS1 (EOD49104.1), ThNIS1 (KKO99972.1), CdNIS1 (ODN99725.1), KhNIS1 (OCF35386.1), SpNIS1 (RSH91236.1), XhNIS1 (TGJ81146.1), PvNIS1 (XP_040715065.1), and DhNIS1 (POS77150.1).

## Supplementary Information

Additional file 1: **Fig. S1.** VmNIS1 requires a signal peptide to induce cell death in *N. benthamiana*. **Fig. S2.** VmNIS2 suppresses VmNIS1-triggered cell death in *N. benthamiana*. **Fig. S3.** Expression of VmNIS1 in *E. coli*. **Fig. S4.** Targeted deletion of *VmNIS1* and *VmNIS2* in *Valsa mali*. **Fig. S5.** *VmNIS1* and *VmNIS2* deletion mutants exhibit normal filamentous growth. **Fig. S6.** *VmNIS1* and *VmNIS2* deletion mutants show no apparent alteration on tolerance to KCl stress. **Fig. S7.** VmNIS1 and VmNIS2 interact with *N. benthamiana* BAK1. **Fig. S8.** Pep13 cannot trigger obvious ROS burst in *N. benthamiana*.

Additional file 2: **Table S1.** Primers used in this study.

## Data Availability

The data that support the findings of this study are included in this article and its supplementary information files.
