# Supplementary material for: Two NIS1-like proteins from apple canker pathogen (Valsa mali) play distinct roles in plant recognition and pathogen virulence
Source: Stress Biol. 2022 Jan 17;2(1):7. doi: 10.1007/s44154-021-00031-0 (PMC10442039; doi:10.1007/s44154-021-00031-0)
Supplement: Supplementary file 1 — Fig. S1. VmNIS1 requires a signal peptide to induce cell death in N. benthamiana. Fig. S2. VmNIS2 suppresses VmNIS1-triggered cell death in N. benthamiana. Fig. S3. Expression of VmNIS1 in E. coli. Fig. S4. Targeted deletion of VmNIS1 and VmNIS2 in Valsa mali. Fig. S5. VmNIS1 and VmNIS2 deletion mutants exhibit normal filamentous growth. Fig. S6. VmNIS1 and VmNIS2 deletion mutants show no apparent alteration on tolerance to KCl stress. Fig. S7. VmNIS1 and VmNIS2 interact with N. benthamiana BAK1. Fig. S8. Pep13 cannot trigger obvious ROS burst in N. benthamiana. [file 44154_2021_31_MOESM1_ESM.docx]

**
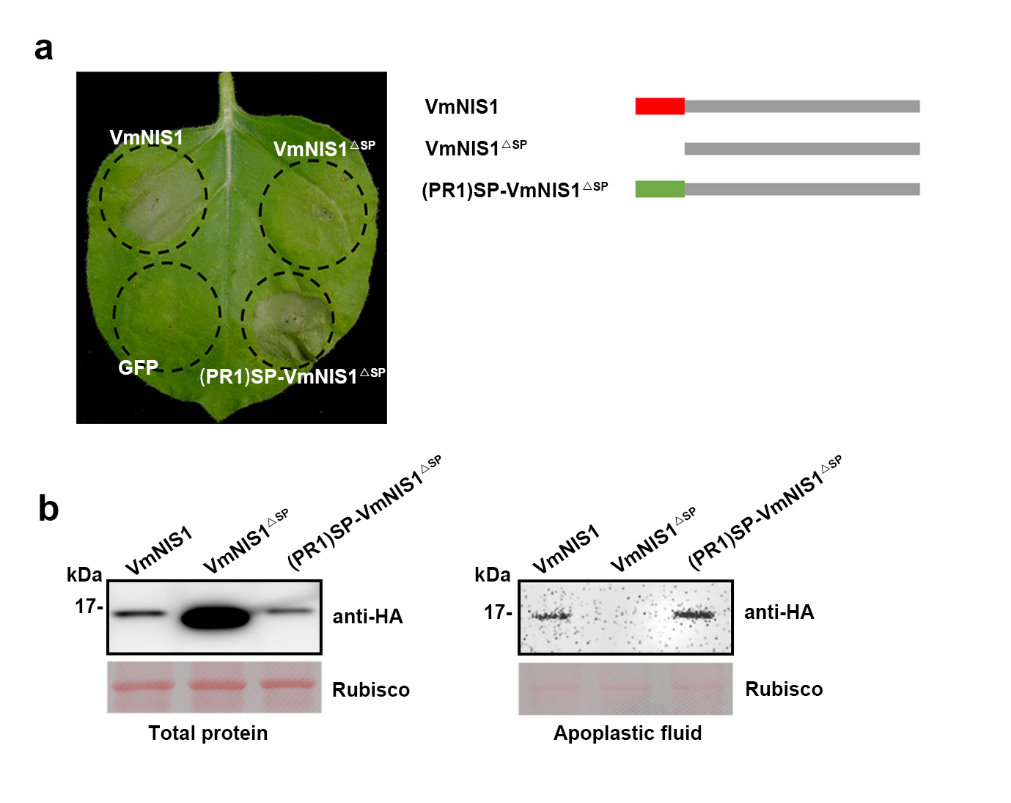
**

**Fig. S1** VmNIS1 requires a signal peptide to induce cell death in *N. benthamiana*. **a** Representative leaves showing cell death induced by VmNIS1 and the fusion variant (PR1)SP-VmNIS1^ΔSP^. VmNIS1, VmNIS1 without signal peptide (VmNIS1^ΔSP^), the fusion variant (PR1)SP-VmNIS1^ΔSP^ and GFP were transiently expressed in *N. benthamiana* leaves by agroinfiltration*.* Photographs were taken 5 days post agroinfiltration (dpa). A diagram showing VmNIS1 constructs was depicted. **b** Western blotting analysis of total proteins or apoplastic fluid extracted from *N. benthamiana* leaves expressing VmNIS1, VmNIS1^ΔSP^ and (PR1)SP-VmNIS1^ΔSP^ with anti-HA antibody. Ponceau S-stained Rubisco protein was shown as a loading control.


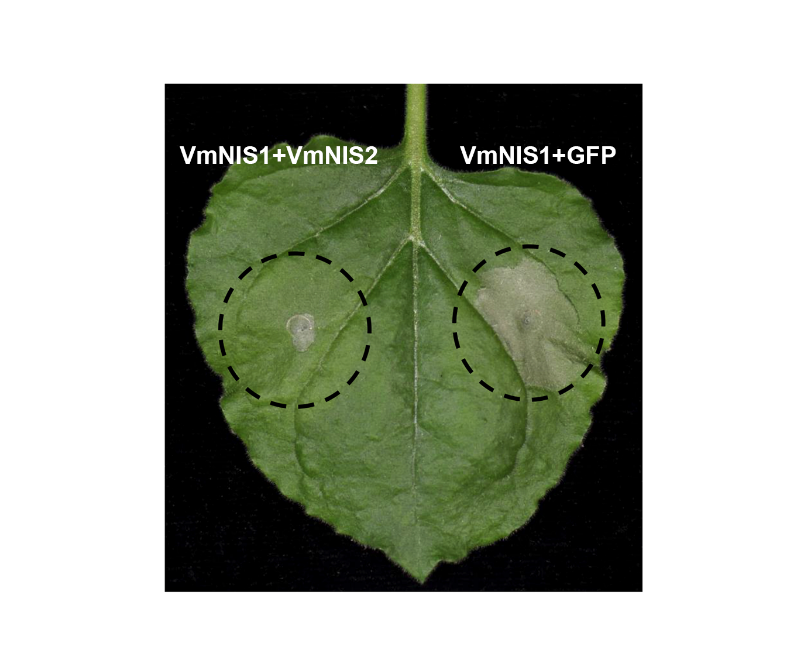


**Fig. S2** VmNIS2 suppresses VmNIS1-triggered cell death in *N. benthamiana*. VmNIS2 and GFP were transiently expressed in *N. benthamiana* leaves via agroinfiltration, and VmNIS1 was agroinfiltrated in the same infiltrated sites 24 h later. Representative leaves were photographed 4 days post agroinfiltration of VmNIS1.


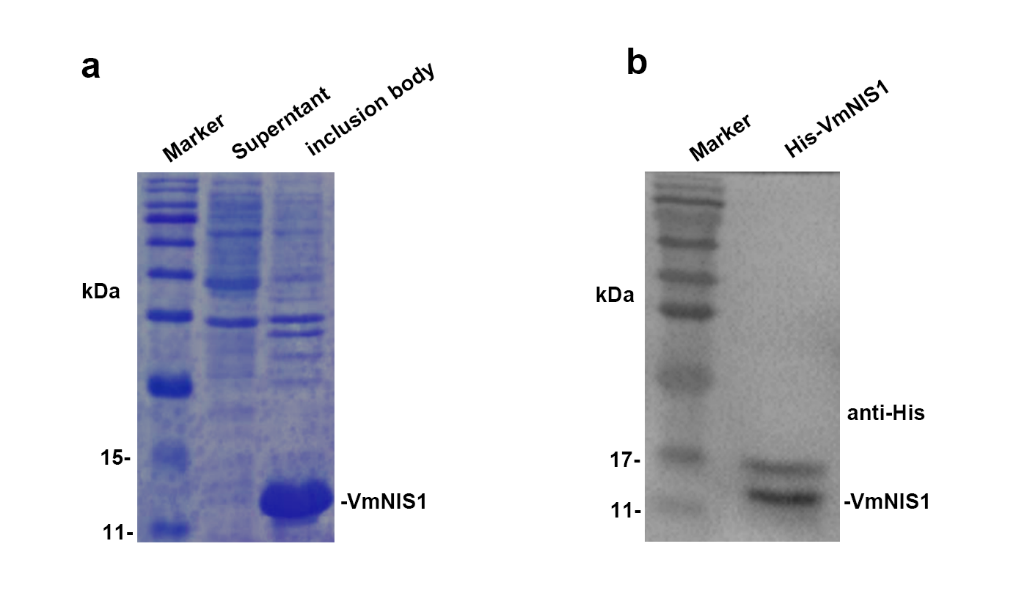


**Fig. S3** Expression of VmNIS1 in *Escherichia coli*. **a** Induced expression of VmNIS1 in *E. coli* strain BL21 (DE3). The proteins samples were fractionated by SDS-PAGE and visualized after Coomassie brilliant blue straining. **b** Western blotting analysis of VmNIS1 purified protein with anti-His antibody.


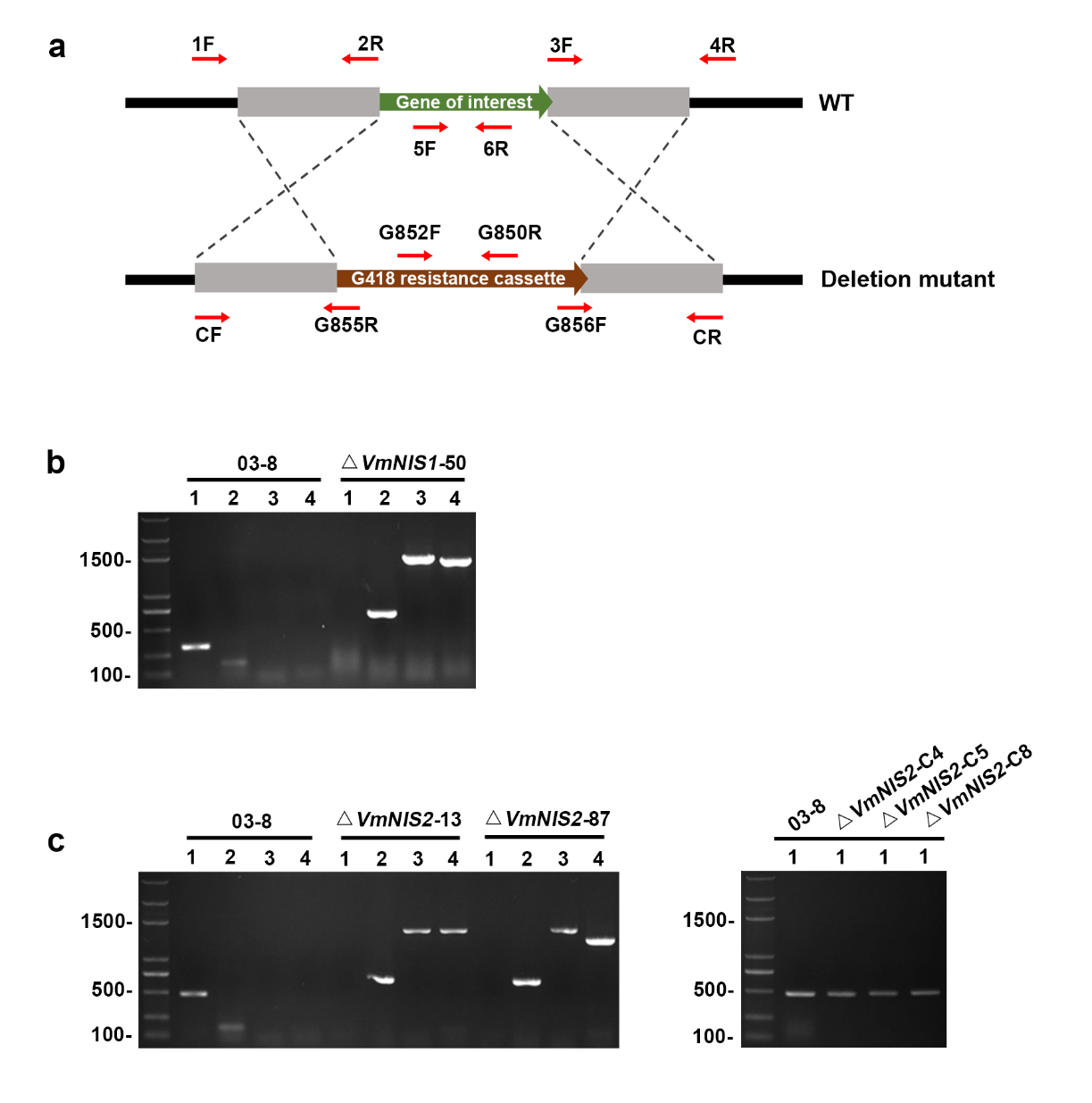


**Fig. S4.** Targeted deletion of *VmNIS1* and *VmNIS2* in *Valsa mali*. **a** Schematic representation showing targeted replacement strategy of *VmNIS1* or *VmNIS2* with G418-resistant cassette. Orientation of primers used to generate gene deletion transformants are indicated by red arrows. **b** Verification of *VmNIS1* deletion mutant (Δ*VmNIS1*-50) by PCR. 1, targeted gene (*VmNIS1*), detected with 5F/6R; 2, G418-resistanct cassette, detected with G852F/G850R; 3, upstream flank, detected with 1F/G855R; 4, downstream flank, detected with G856F/4R. **c** Verification of *VmNIS2* deletion mutants and complementation transformants by PCR. 1, targeted gene (*VmNIS2*), detected with 5F/6R; 2, G418-resistanct cassette, detected with G852F/G850R; 3, upstream flank, detected with 1F/G855R; 4, downstream flank, detected with G856F/4R.


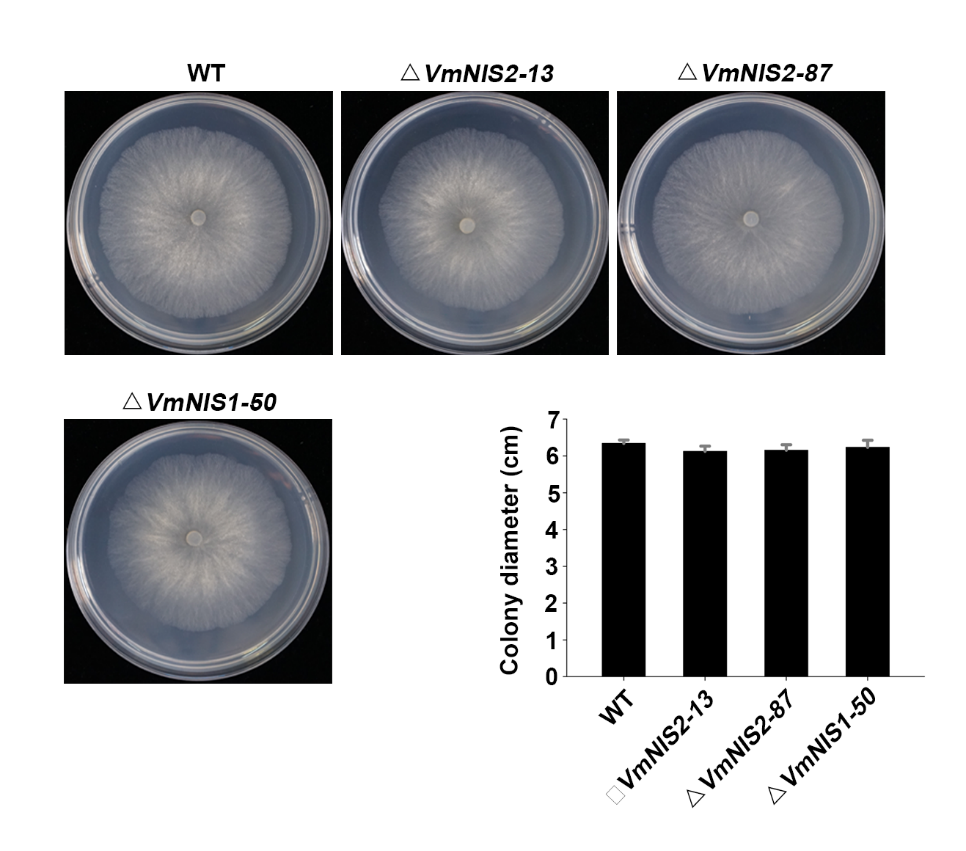


**Fig. S5** *VmNIS1* and *VmNIS2* deletion mutants exhibit normal filamentous growth. *VmNIS1* deletion mutant (Δ*VmNIS1*-50), *VmNIS2* deletion mutants (Δ*VmNIS2*-13 and Δ*VmNIS2*-87), and *V. mali* wild type strain 03-8 were cultured on PDA plates in the dark at 25°C. Representative photographs were taken and colony diameters were calculated 48 h later. Values represent the means ± SD.


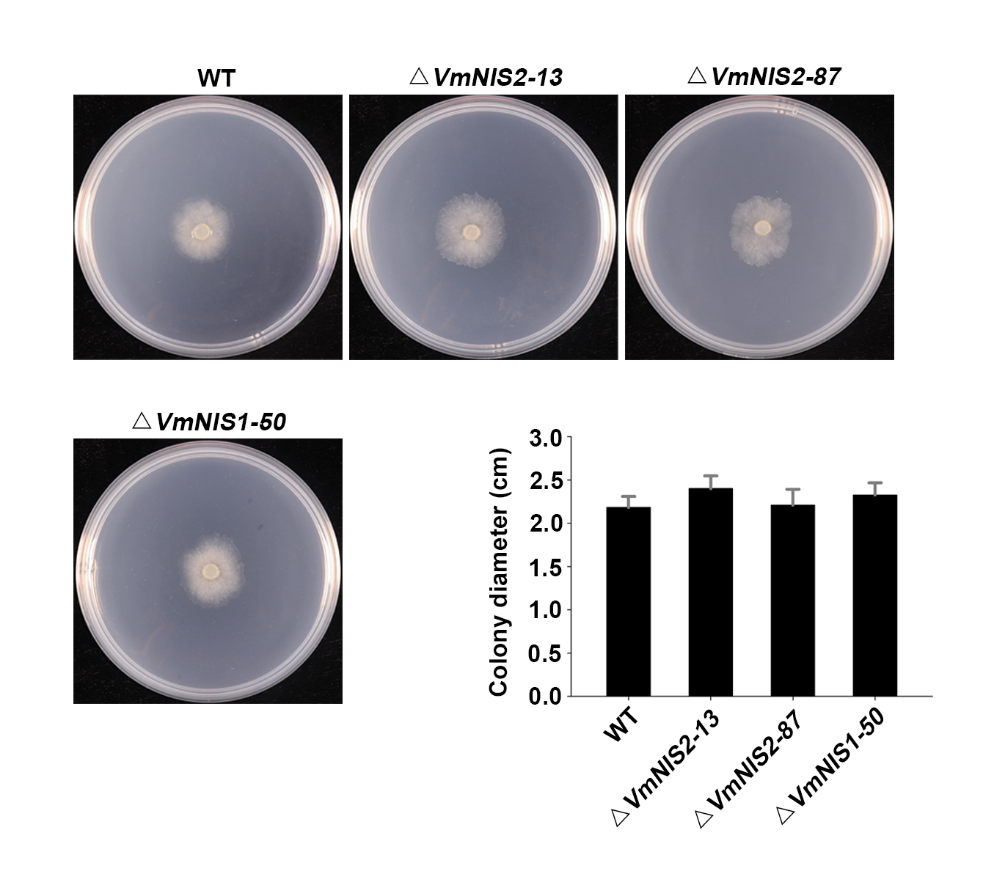


**Fig. S6** *VmNIS1* and *VmNIS2* deletion mutants show no apparent alteration on tolerance to KCl stress. *VmNIS1* deletion mutant (Δ*VmNIS1*-50), *VmNIS2* deletion mutants (Δ*VmNIS2*-13 and Δ*VmNIS2*-87), and *V. mali* wild type strain 03-8 were cultured on PDA plates supplemented with 500 mM KCl, at 25°C under dark conditions. Representative photographs were taken 48 h later. Values represent the means ± SD from nine biological replicates.

**
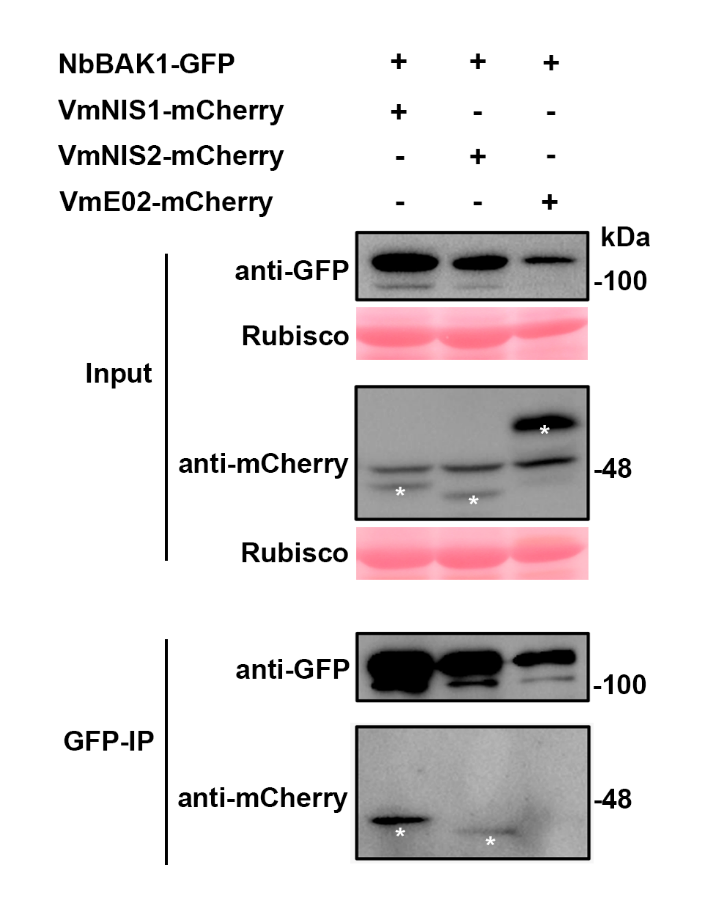
**

**Fig. S7** VmNIS1 and VmNIS2 interact with *N. benthamiana* BAK1. *N. benthamiana* leaves co-expressing GFP-tagged BAK1 and mCherry-tagged VmNIS1, VmNIS2 or VmE02 were harvested 36 h post agroinfiltration. Total proteins were extracted and immunoprecipitated with GFP-Trap A beads. Proteins samples were subjected to western blotting detection using anti-GFP and anti-mCherry antibodies, respectively. Ponceau S-stained Rubisco protein was shown as a loading control. White asterisks indicate the protein bands that correspond to VmNIS1, VmNIS2 or VmE02.

**
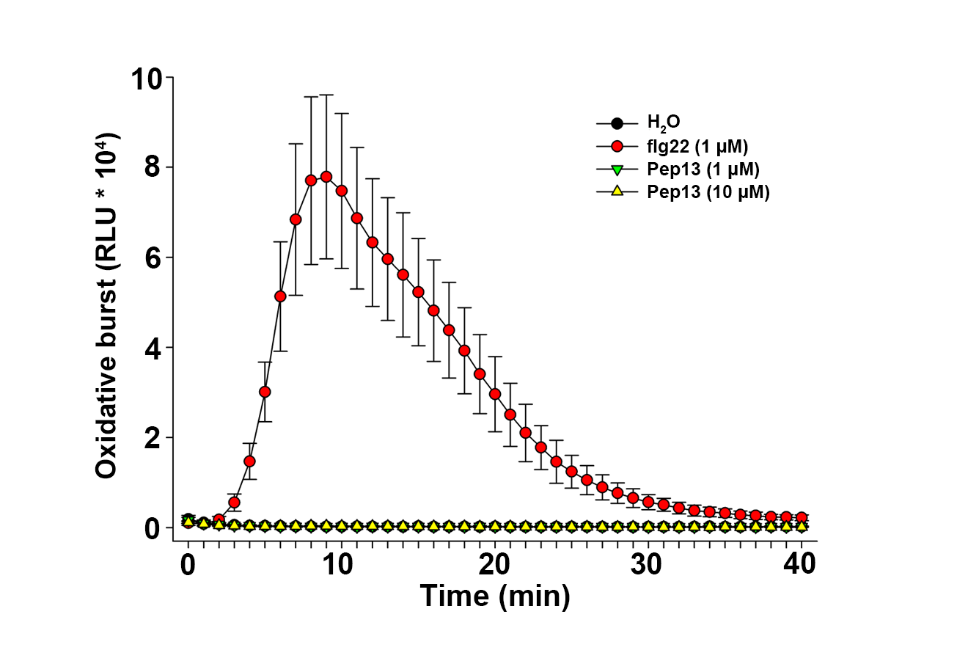
**

**Fig. S8** Pep13 cannot trigger obvious ROS burst in *N. benthamiana*. ROS production represented by relative luminescence units (RLU) was measured through treatment with 1 μM flg22 peptide, 1 μM Pep13 peptide or 10 μM Pep13 peptide.
